# Viral vector-mediated selective and reversible blockade of the pathway for visual orienting in mice

**DOI:** 10.3389/fncir.2013.00162

**Published:** 2013-10-11

**Authors:** Thongchai Sooksawate, Kaoru Isa, Ryosuke Matsui, Shigeki Kato, Masaharu Kinoshita, Kenta Kobayashi, Dai Watanabe, Kazuto Kobayashi, Tadashi Isa

**Affiliations:** ^1^Department of Developmental Physiology, National Institute for Physiological SciencesOkazaki, Japan; ^2^Department of Pharmacology and Physiology, Faculty of Pharmaceutical Sciences, Chulalongkorn UniversityBangkok, Thailand; ^3^Department of Molecular and Systems Biology, Graduate School of Biostudies, Kyoto UniversityKyoto, Japan; ^4^Department of Molecular Genetics, Institute of Biomedical Sciences, Fukushima Medical University School of MedicineFukushima, Japan; ^5^Section of Viral Vector Development, National Institute for Physiological SciencesOkazaki, Japan; ^6^Department of Life Sciences, The Graduate University for Advanced Studies (SOKENDAI)Hayama, Japan

**Keywords:** superior colliculus, pontine reticular formation, orienting behavior, viral vector, Tet-ON, tetanus neurotoxin, mouse

## Abstract

Recently, by using a combination of two viral vectors, we developed a technique for pathway-selective and reversible synaptic transmission blockade, and successfully induced a behavioral deficit of dexterous hand movements in macaque monkeys by affecting a population of spinal interneurons. To explore the capacity of this technique to work in other pathways and species, and to obtain fundamental methodological information, we tried to block the crossed tecto-reticular pathway, which is known to control orienting responses to visual targets, in mice. A neuron-specific retrograde gene transfer vector with the gene encoding enhanced tetanus neurotoxin (eTeNT) tagged with enhanced green fluorescent protein (EGFP) under the control of a tetracycline responsive element was injected into the left medial pontine reticular formation. 7–17 days later, an adeno-associated viral vector with a highly efficient Tet-ON sequence, rtTAV16, was injected into the right superior colliculus. 5–9 weeks later, the daily administration of doxycycline (Dox) was initiated. Visual orienting responses toward the left side were impaired 1–4 days after Dox administration. Anti-GFP immunohistochemistry revealed that a number of neurons in the intermediate and deep layers of the right superior colliculus were positively stained, indicating eTeNT expression. After the termination of Dox administration, the anti-GFP staining returned to the baseline level within 28 days. A second round of Dox administration, starting from 28 days after the termination of the first Dox administration, resulted in the reappearance of the behavioral impairment. These findings showed that pathway-selective and reversible blockade of synaptic transmission also causes behavioral effects in rodents, and that the crossed tecto-reticular pathway clearly controls visual orienting behaviors.

## Introduction

To study structure-function relationships within neural circuits, it is necessary to manipulate the activity of an “identified” population of neuronal elements in these circuits. Recent advances in molecular genetic techniques enabled such targeted manipulation by making transgenic animals with cell-specific promoters (Kobayashi et al., 1995; Watanabe et al., 1998). However, such techniques have generally been constrained to those animals in which the transgenic manipulation of gene expression is possible (i.e., mouse, nematode, *Drosophila*, etc.) and to neuron types whose cell-specific promoter has been identified. An alternative possibility is to use viral vectors to introduce particular genes. To enable pathway specificity, lentivirus vectors pseudotyped with rabies virus glycoprotein (RVG) were developed as vectors specific for retrograde transport (Kato et al., 2007). However, such vectors were not sufficiently efficient to affect enough of a specific neuron population to induce behavioral effects, especially in larger animals such as non-human primates. To overcome such difficulties, Kobayashi and colleagues recently enhanced the retrograde transport efficiency of lentivirus vectors by pseudotyping with a chimera of rabies virus and vesicular stomatitis virus glycoprotein (VSVG) [highly efficient retrograde gene transfer (HiRet) vector; Kato et al., 2011a]. We incorporated a tetracycline responsive element (TRE) and enhanced tetanus neurotoxin (eTeNT) tagged with enhanced green fluorescent protein (EGFP) into this retrograde vector (HiRet-TRE-EGFP.eTeNT). We also incorporated the newly developed efficient Tet-ON sequence rtTAV16 into an adeno-associated type 2 virus vector (AAV-2) with a cytomegalovirus promoter (CMV) (AAV2-CMV-rtTAV16) (Kinoshita et al., 2012) as a switch to regulate the first construct. To study the function of a subpopulation of propriospinal neurons (PNs) whose cell bodies are located in the mid-cervical segments and project to hand/arm motor neurons in macaque monkeys, we injected HiRet-TRE-EGFP.eTeNT into the ventral horn of the C6–Th1 segments; one week later, we injected AAV2-CMV-rtTAV16 into the intermediate zone of the C2–C5 segments where the PN cell bodies are located. 1–2 months later, we initiated the administration of doxycycline (Dox). We could observe deficits in reach and grasp movements. Acute electrophysiological experiments clarified that approximately 90% of the transmission through the PNs was blocked. Theoretically, this method should be available for universal use in the various pathways of the central nervous system without the need to identify cell-specific promoters or develop genetically modified animals.

At the current stage, we still need to know the fundamental properties of this technique and how to optimize it. For this purpose, however, non-human primates are not the best animal species because the number of available animals is limited, and behavioral and histological analyses are time consuming. Therefore, we decided to use mice to establish the fundamental protocol of this technique. Moreover, we wished to select a pathway whose function is easy to investigate using behavioral observations. We chose the crossed tectoreticular pathway (cTRNs), which originates from the intermediate and deep layers of the midbrain superior colliculus (SC) and terminates in the medial pontomedullary reticular formation contralateral to the SC (Grantyn and Berthoz, 1987; Redgrave et al., 1990; Isa and Sasaki, 2002; Sooksawate et al., 2005, 2008). This pathway is known to control the orienting response of the eyes, head, and body to visual targets (Wurtz and Albano, 1980; Sparks, 1986; Dean et al., 1989; Isa and Sasaki, 2002; Isa and Hall, 2009), which is an innate behavior that does not require training. In this study, we focused on the time course of the effect of pathway blockade through behavioral and histological examinations after starting Dox administration and after its offset. We also tested the reversibility of the effects by halting and repeating Dox administration. This technique proved very feasible in mice, suggesting that it could be used universally as a relatively convenient method for the pathway-specific manipulation of neural activity and gene expression.

## Materials and methods

**Figure 1:**
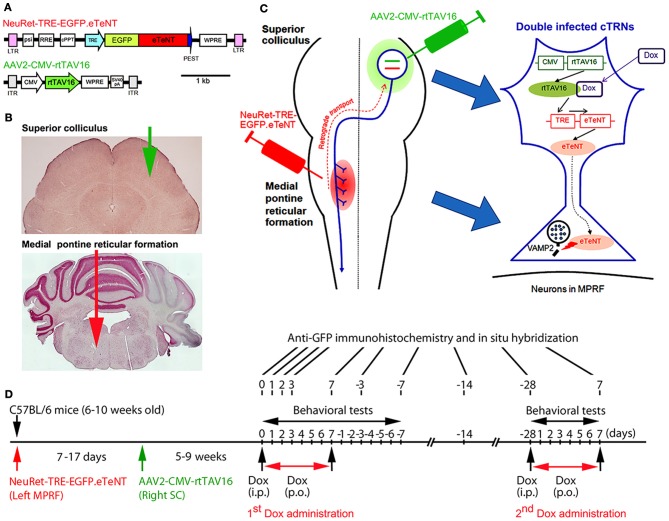
**Experimental protocols for the selective blockade of the crossed tectoreticular pathway by double infection with the NeuRet lentiviral vector (carrying EGFP.eTeNT) and the AAV2 vector (carrying the Tet-ON sequence, CMV-rtTAV16). (A)** The design of the two viral vectors. **(B)** Photomicrographs of the superior colliculus (SC) and medial pontine reticular formation (MPRF) showing the viral vector injection targets. **(C)** Diagram for the double injection of the viral vectors into the MPRF and SC, and the interaction of NeuRet-TRE-EGFP.eTeNT and AAV2-CMV-rtTAV16 in the double infected crossed tectoreticular neurons (cTRNs). **(D)** The experimental schedule of this study.

The animal experimental procedures in this study were conducted in accordance with the Guidelines of the National Institutes of Health and the Ministry of Education, Culture, Sports, Science, and Technology of Japan, and were approved by the Institutional Animal Care and Use Committee of the National Institutes of Natural Sciences. All attempts were made to minimize the suffering and number of animals used in this study. The methods and time schedule of the present experiments are summarized in Figure 1.

### Preparation of viral vectors

Vector preparation was described by Kinoshita et al. (2012). However, a major difference was that we used a neuron-specific highly efficient retrograde gene transfer lentivirus (NeuRet) vector (Kato et al., 2011b) instead of the HiRet vector, because the NeuRet vector is specifically taken up by axons at the injection site.

#### NeuRet-TRE-EGFP.eTeNT

The NeuRet vector is a pseudotype of a human immunodeficiency virus type 1-based lentiviral vector with fusion glycoprotein type C. In previous studies, it has been shown that pseudotyping the lentiviral vector with the RVG changed the property of the vector specific for retrograde gene transfer (Kato et al., 2007). However, the efficiency of the gene transfer was not enough to affect the majority of neurons projecting to the injection site. Later, it was found that replacing RVG with a chimeric protein of RVG and VSVG domains greatly enhanced the efficiency of retrograde gene transfer (highly efficient retrograde gene transfer vector or HiRet vector) (Kato et al., 2011a). Then, the use of a different chimeric protein composing RVG and VSVG (fusion glycoprotein type C) switched the properties to neuron-specific infection (NeuRet) (Figure 1A; Kato et al., 2011b). In the present study, the envelope plasmid encoding fusion glycoprotein type C, which was under the control of the cytomegalovirus (CMV) enhancer/chicken β-actin promoter, was used for vector production. Chimeric EGFP.eTeNT.PEST was generated by fusing the human codon-optimized tetanus neurotoxin light chain (eTeNT) with the EGFP derived from pEGFP-N1 vector (Clontech, Mountain View, CA, USA) and the PEST sequence of ornithine decarboxylase, as reported previously (Kinoshita et al., 2012). The transfer plasmid pLV-TRE-EGFP.eTeNT.PEST was based on pFUGW (a gift from D. Baltimore, California Institute of Technology, Pasadena, CA, USA), and constructed by swapping the ubiquitin promoter-EGFP sequence with the TRE/CMV promoter of the pTRE-Tight vector (Clontech, Mountain View, CA, USA) and EGFP.eTeNT.PEST. The NeuRet vector encoding EGFP.eTeNT was placed downstream of the TRE promoter (termed NeuRet-TRE-EGFP.eTeNT) and was prepared as described previously (Inoue et al., 2012).

#### AAV2-CMV-rtTAV16

The rtTA variant rtTAV16 was generated by introducing the V9I, G12S, F67S, F86Y, R171K, and A209T mutations into rtTA2S-M2 of the pTet-ON advanced vector (Clontech, Mountain View, CA, USA), as reported previously (Kinoshita et al., 2012). Plasmid pAAV2-CMV-rtTAV16 (Figure 1A) is based on pAAV-MCS (Agilent Technologies, Inc., Santa Clare, CA, USA), and was constructed by inserting the CMV promoter sequence of the pTet-On advanced vector rtTAV16, the woodchuck hepatitis virus posttranscriptional regulatory element WPRE sequence of pFUGW, and the SV40 polyadenylation signal (SV40pA) of the pCMV-script vector (Agilent Technologies, Tokyo, Japan) into a multiple cloning site. The AAV vector for *in vivo* injection was produced as described previously (Kaneda et al., 2011).

### Injections of NeuRet-TRE-EGFP.eTeNT and AAV2-CMV-rtTAV16

We anesthetized 6–10-week-old male C57BL/6 mice with an intraperitoneal injection of a mixture of ketamine (60 mg/kg body weight) and xylazine (10 mg/kg body weight). In addition, dexamethasone (5.5 mg/kg body weight) was injected intramuscularly as premedication. The head of the mouse was fixed to the stereotaxic apparatus (Narishige, Tokyo, Japan) and injections of the vectors were made from the dorsal approach. NeuRet-TRE-EGFP.eTeNT (0.8–1.2 μL); titer, 3.3–12.1 × 10^11^ copies/mL) was injected into the medial pontine reticular formation (MPRF) on the left side (Figures 1B,C) using a thin glass micropipette (tip diameter, 50–70 μm) inclined by 45°caudally to the vertical axis of the stereotaxic coordinates (Franklin and Paxinos, 2008), −8.3 mm from the bregma, 0.7 mm lateral to the midline, and at 3.6 and 4.4 mm from the presumed dorsal surface of the cerebellar cortex (0.4–0.6 μL/point of injection). 7–17 days after the NeuRet-TRE-EGFP.eTeNT injection, AAV2-CMV-rtTAV16 (0.8–1.0 μL; titer, 1.96 × 10^13^ particles/mL) was injected into the SC on the right side (Figures 1B,C). A small hole was made in the skull over the occipital cortex and a thin glass micropipette (tip diameter; 50–70 μm) was inserted vertically into the right SC, −4.0 to −4.2 mm from the bregma, 1.1–1.2 mm lateral to the midline, and at 1.1–1.6 and 1.4–2.3 mm from the presumed dorsal surface of the cerebral cortex (0.4–0.5 μL/point of injection). We used a syringe pump (ESP-32; Eicom, Kyoto, Japan) for the injection; the injection rate was 0.1 μL/min. Before removing the glass micropipette from the injection site, we waited for 5 min.

The transfer plasmid pLV-TRE-EGFP.eTeNT.PEST is switched on to produce the tetanus neurotoxin only when the rtTAV16 sequence provided by the AAV2-CMV-rtTAV16 vector is expressed in the same neuron and activated by Dox (Figure 1C). The tetanus neurotoxin blocks the transmitter release by cleaving VAMP-2 at the nerve terminals, but does not kill the cell (for review see Montecucco and Schiavo, 1994).

### Dox administration

5–9 weeks after the injection of AAV2-CMV-rtTAV16, the daily administration of Dox was initiated by a single intraperitoneal injection (10 μg/g body weight) of Dox in a 0.9% NaCl solution, followed by the continuous oral administration of Dox in the drinking water (3 mg/mL in a 5% sucrose solution) for 7 days (Figure 1D). In some animals, the second period of Dox administration (2nd Dox administration) was conducted from 28 days after the offset of the first period of Dox administration (1st Dox administration). One group of mice received Dox continually for 21 days for histological analysis.

### Behavioral tests for visual orienting and turning behavior

We utilized three tests to assess visual orienting and turning behaviors: (1) the visual placing response, (2) the visual orienting response, and (3) a turning behavior test (Figure 1D). The behavioral tests began on the day before Dox administration, continued during Dox administration for 7 days, and after Dox administration for 7 days. All tests were performed between 6.00 AM and 3.00 PM.

#### Visual placing response

**Figure 2:**
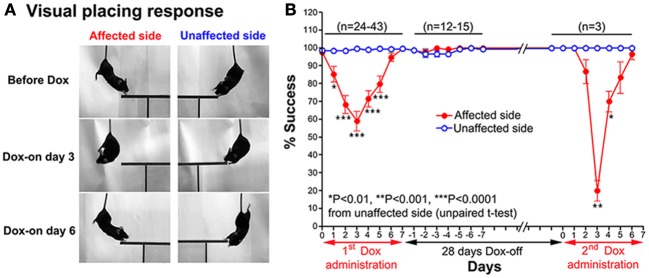
**(A)** Series of photographs showing the effect of the Tet-induced tetanus neurotoxin-expressing system on the visual placing response toward the affected side (Dox-on day 3) and disappearance of the effect (Dox-on day 6), compared with the unaffected side. **(B)** Effect of the Tet-induced tetanus neurotoxin-expressing system on the visual placing response toward the affected side after the 1st Dox administration for 7 days, termination of Dox administration for 28 days, and the 2nd Dox administration for 7 days.

A modified visual placing response test was used to evaluate visual orienting behavior toward the affected and unaffected sides of the tested mouse. This test was modified from Metz and Schwab (2004) and Pinto and Enroth-Cugell (2000). In this test, the mouse was suspended by holding its tail and then lowered toward a plastic plate either on the left or right side of the head without any contact to the vibrissae. Normally, when the head of a mouse was lowered to near the edge of the plastic plate, it turned its head and trunk, and extended its forelimbs to place them on the plate (see Figure 2A). The procedure was conducted bilaterally (10 trials per side each day). The number of times the mouse successfully placed its forelimbs on the plate was counted.

#### Visual orienting response

**Figure 3:**
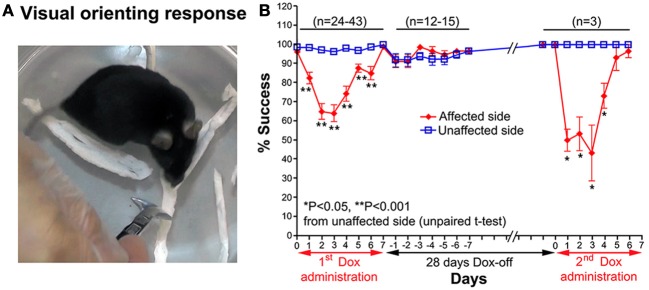
**(A)** A photograph showing the visual orienting response of a mouse toward the unaffected side. **(B)** Effect of the Tet-induced tetanus neurotoxin-expressing system on the visual orienting response of the affected side after the 1st Dox administration for 7 days, termination of Dox administration for 28 days, and the 2nd Dox administration for 7 days.

A visual orienting response test was used to evaluate visual orienting behavior toward a visual target presented either on the affected or unaffected side of the tested mouse. The mouse was placed inside a small glass cylinder (15 cm height, 14 cm inner diameter), and a stimulus (a small long-handle stainless steel chemical spoon) was quickly moved into the visual field of the mouse from behind, in the same horizontal plane as its eyes, until the stimulus was located between its eyes (Smith et al., 1998). Normally, when the stimulus entered its visual field, the mouse turned its head toward the stimulus (Figure 3A). The procedure was conducted bilaterally in a pseudorandom sequence (10 trials per side each day). The number of times the mouse successfully oriented itself to the stimulus was counted.

#### Turning behavior

**Figure 4:**
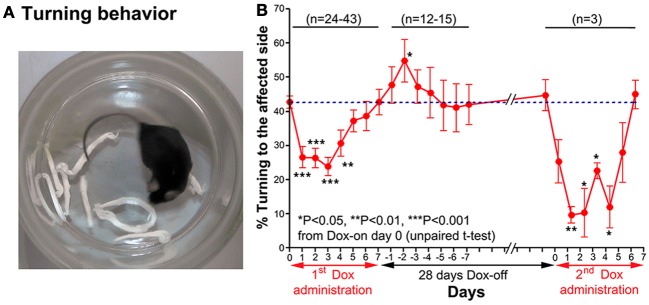
**(A)** A photograph showing the turning behavior of a mouse to the unaffected side (right). **(B)** Effect of the Tet-induced tetanus neurotoxin-expressing system on turning behavior after the 1st Dox administration for 7 days, termination of Dox administration for 28 days, and the 2nd Dox administration for 7 days.

A turning behavior test was used to evaluate the effect of the Tet-induced tetanus neurotoxin-expressing system on the preferred side of turning of the tested mouse after Dox administration. The mouse was placed into a small glass cylinder (15 cm height, 14 cm inner diameter), which forced it to turn left or right inside the cylinder (Figure 4A). The number of complete turns (360°) to each side was recorded for 10 min each day.

### Immunohistochemical assessments

At the end of the experiments, the mice were deeply anesthetized with an intraperitoneal injection of sodium pentobarbital (80 mg/kg body weight) (Hospira Inc., Lake Forest, IL, USA) and transcardially perfused with 0.05 M phosphate-buffered saline and then 4% paraformaldehyde in a 0.1 M phosphate buffer (pH 7.4). The brainstem and spinal cord were cryoprotected and sectioned at a thickness of 40 μm using a sliding microtome (HM 450; Microm, Walldorf, Germany). To obtain a clearer image of the cell bodies, dendrites and axons of the double infected neurons, we adopted permanent visualization of EGFP-positive cells with anti-GFP immunohistochemistry. In particular, the permanent visualization technique made the axon tracing much easier. Moreover, the distribution of neurons infected by AAV2-CMV-rtTAV16 was assessed with *in situ* hybridization to detect the rtTA sequence (the antisense probe for the tetracycline transactivator, tTA, was a gift from T. Yamamori, National Institute for Basic Biology, Okazaki, Japan). The technical details of the anti-GFP immunohistochemistry and *in situ* hybridization against the rtTAV16 sequence were described previously (Kinoshita et al., 2012) and are available on the web (http://www.nibb.ac.jp/brish/indexE.html), respectively. The number of GFP-positive neurons and tracings of the axons originating from their cell bodies to their target areas were counted and drawn using a camera lucida attached to a light microscope (BX51; Olympus, Tokyo, Japan). Photomicrographs of the histological slices were taken using light microscopes (Axioplan2; Zeiss, Göttingen, Germany and BZ-9000; Keyence, Elmwood Park, NJ, USA).

### Data analysis

Data are expressed as the mean ± standard error of the mean. Significance was tested by Student’s *t*-test, and a *P* value of less than 0.05 was considered to be significant.

## Results

A total of 61 C57BL/6 mice were used in this study; the two viral vectors were injected into 56 mice. As controls, some mice received an injection of NeuRet-TRE-EGFP.eTeNT without Dox administration (*n* = 2), NeuRet-TRE-EGFP.eTeNT and AAV2-CMV-rtTAV16 without Dox administration (*n* = 2), or NeuRet-TRE-EGFP.eTeNT with Dox administration (*n* = 3), and one group of mice was not injected (*n* = 5). No clear behavioral effects could be observed in any of these control groups. Specifically, the % success rate for the visual placing response on the side contralateral to the collicular injection ranged from 95.00 ± 5.00 to 100.0 ± 0.00%, which was not significantly different from the side ipsilateral to the collicular injection (100.0 ± 0.00%). The % success rate for the visual orienting responses on the side contralateral to the collicular injection ranged from 95.00 ± 5.00 to 100.0 ± 0.00%, which was also not significantly different from the side ipsilateral to the collicular injection that ranged from 95.00 ± 5.00 to 100.0 ± 0.00%. Moreover, the % turning rate to the side contralateral to the collicular injection was not significantly different from the side ipsilateral to the collicular injection on any of the testing days (Dox-on day 0 to Dox-on day 7). It ranged from 44.60 ± 5.63 to 50.40 ± 3.15%.

### Behavioral observations

#### Visual placing response

After Dox administration, the visual placing response toward the affected side (left side) of the double infected mice was impaired from Dox-on day 1 to day 5, with the maximal impairment on Dox-on day 3 compared with the unaffected side (right side). The mice failed to turn their head and trunk and place their forelimbs onto the plastic plate when they were lowered with the plastic plate on the affected side (Figure 2A). The % success rate for placing toward the affected side was reduced to 58.95 ± 5.41%, compared with 99.47 ± 0.3671% for the unaffected side (*P* < 0.0001, *n* = 38, unpaired *t*-test; Figure 2B). However, this behavioral effect was gradually reduced from Dox-on day 4 and completely disappeared on Dox-on days 6–7. After the termination of Dox administration, no behavioral effect could be seen on the affected and unaffected sides for 7 days (Dox-off days −1 to −7).

#### Visual orienting response

Impairment of the orienting response could also be found in the visual orienting response test. After Dox administration, the visual orienting response toward the affected side (left side) was impaired from days 1–6, while it was not impaired toward the unaffected side (right side) (Figure 3A). The % success rate of the affected side was reduced to 64.21 ± 4.473%, compared to the 96.32 ± 0.793% of the unaffected side (*P* < 0.001, *n* = 38, unpaired *t*-test; Figure 3B). The behavioral effect was gradually reduced from Dox-on day 4, and completely disappeared by Dox-on day 7. After the termination of Dox administration, no behavioral effect could be seen on the affected and unaffected sides for 7 days (Dox-off days −1 to −7).

#### Turning behavior

Before Dox administration and in the control groups, the mice almost equally preferred to turn toward the left and right. However, after Dox administration, the mice preferred to turn to the unaffected side (right) (Figure 4A). The % turning to the affected side (left side) was reduced from Dox-on days 1 to 4, with the maximal reduction on day 3 (Figure 4B). The % turning to the affected side was reduced to 23.92 ± 2.721% from the 42.77 ± 1.824% of the controls on day 0 (*P* < 0.001, *n* = 38, unpaired *t*-test). Then, the % turning to the affected side gradually returned to the same level as before Dox administration by Dox-on day 7. It is noteworthy that after the termination of Dox administration, % turning to the affected side (left side) increased to its highest level on Dox-off day −2 (*P* < 0.05, *n* = 15, unpaired *t*-test), before returning to the same level as before Dox administration on Dox-off days −5 to −7.

### Reversibility of the dox-on effects

28 days after the termination of the 1st Dox administration (for 7 days), the 2nd Dox administration was started for another 7 days. All of the behavioral tests for visual orienting were found to be impaired after the 2nd Dox administration. The pattern and time course of the impairments were almost the same as for the 1st Dox administration. The % success rates for the visual placing response (Figure 2B) and visual orienting response (Figure 3B) and % turning (Figure 4B) toward the affected side were reduced on Dox-on days 1–2 to days 4–5 and, then, gradually increased to the same levels as before Dox administration on days 6–7. Although, the maximal effects of the 2nd Dox administration on all behavioral tests appeared to be greater, they were not significantly different from the 1st Dox administration (visual placing response, *P* = 0.0531; visual orienting response, *P* = 0.2123; turning behavior, *P* = 0.1544, unpaired *t*-test). These results indicate that the selective blockade of the crossed tectoreticular pathway by double infection with NeuRet-TRE-EGFP.eTeNT and AAV2-CMV-rtTAV16 can be performed repeatedly with an interval of 28 days.

### Histological analysis

**Figure 5:**
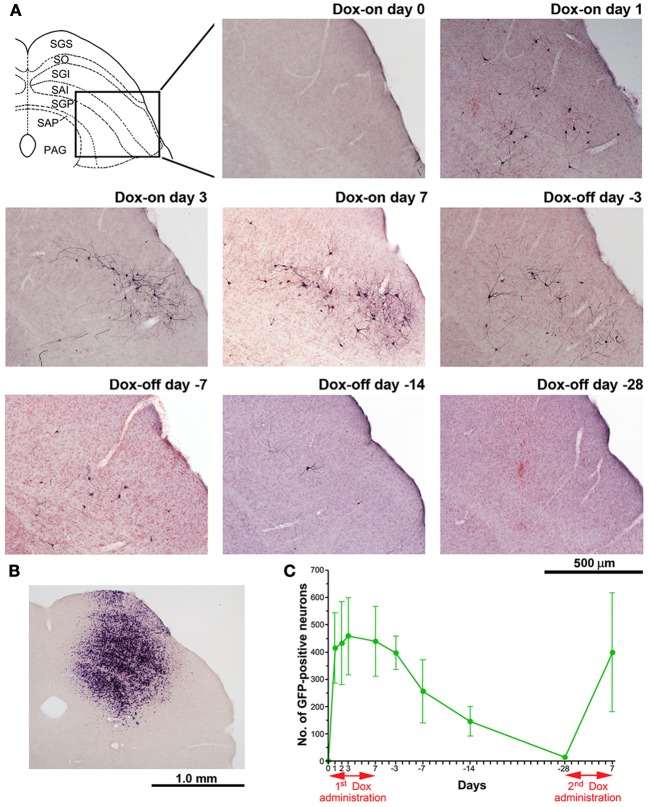
**Photomicrographs of GFP-positive cTRNs and *in situ* hybridization to visualize rtTAV16 in the superior colliculus (SC) of double infected mice. (A)** Schematic diagram of the laminar structure of the mouse SC (frontal plane) and photomicrographs of the SC slices processed with anti-GFP immunohistochemistry (frontal section, inset area in the SC diagram), before Dox administration (Dox-on day 0), Dox administration for 1 day (Dox-on day 1), 3 days (Dox-on day 3), 7 days (Dox-on day 7), and after the termination of Dox administration for 3 days (Dox-off day −3), 7 days (Dox-off day −7), 14 days (Dox-off day −14), and 28 days (Dox-off day −28). **(B)** *In situ* hybridization against the tTA sequence in an SC slice indicating the cells infected by AAV2. **(C)** Average number of GFP-positive neurons in the SC after the 1st Dox administration for 7 days, termination of Dox administration for 28 days, and after the 2nd Dox treatment for 7 days (*n* = 3). Abbreviations: SGS, superficial gray layer; SO, optic layer; SGI, intermediate gray layer; SAI, intermediate white layer; SGP, deep gray layer; SAP, deep white layer; PAG, periaqueductal gray.

The number of GFP-positive crossed tectoreticular neurons (cTRNs) in the intermediate and deep layers of the SC of the double infected mice increased sharply after the start of Dox administration for 1 day and reached its maximum on Dox-on day 3 (Figures 5A,C). The maximum number of GFP-positive cTRNs was 458.8 ± 141.4 neurons (*n* = 6). After Dox administration was terminated, the number of GFP-positive cTRNs was gradually reduced on Dox-off days −3 to −14, and had returned to the baseline level by Dox-off day −28. To confirm the injection site of AAV2 in the right SC, *in situ* hybridization of rtTAV16 was performed. The injection sites were found to be located in the intermediate to deep layers of the right SC (Figure 5B). To demonstrate the reversibility of the Dox-on effects in these doubled infected mice, the 2nd Dox administration was started after a 28-day Dox-off period. The number of GFP-positive cTRNs in the intermediate and deep layers of the right SC increased again to 400.0 ± 218.3 (*n* = 3; Figure 5C). The histological results paralleled the impairment of visual orienting behavior after the 2nd Dox treatment in this group (Figures 2B, 3B, 4B).

**Figure 6:**
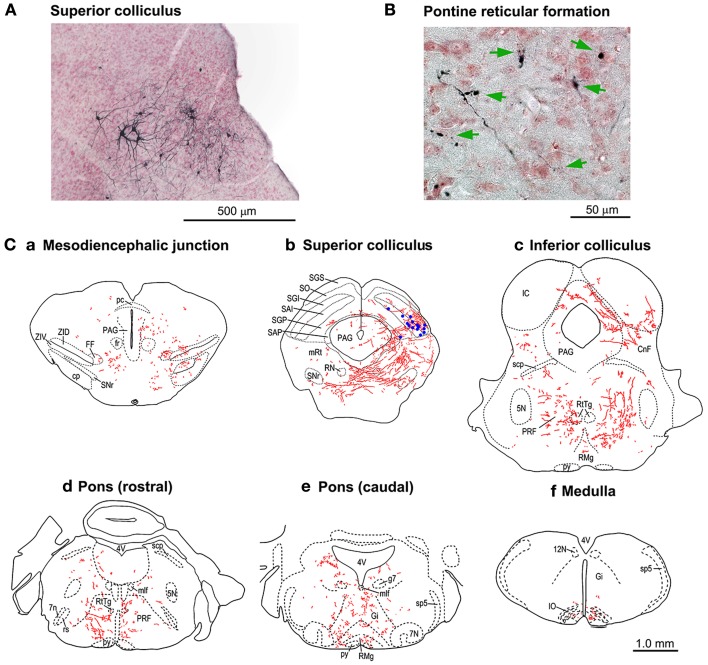
**Tracings of the axonal trajectories of the GFP-positive cTRNs in the superior colliculus (SC) in the mice with Dox treatment for 21 days. (A)** GFP-positive cTRNs in the right SC. **(B)** Axon terminals (arrows) in the left pontine reticular formation. **(C)** Tracings of the axons and their terminals in the brainstem: a. Pretectum; b. Midbrain (SC level); c. Midbrain (inferior colliculus level); d. Pons (rostral part); e. Pons (caudal part); f. Medulla. GFP-positive cell bodies are indicated as blue dots in Cb. Abbreviations: 5N, trigeminal nucleus; 7N, facial nucleus; 7n, facial nerve; 12N, hypoglossal nucleus; CnF, cuneiform nucleus; cp, cerebral peduncle; FF, fields of Forel; fr, fasciculus retroflexus; g7, genu of the facial nerve; Gi, gigantocellular reticular nucleus; IC, inferior colliculus; IO, inferior olive; mlf, medial longitudinal fasciculus; mRt, mesencephalic reticular formation; PAG, periaqueductal gray; pc, posterior commissure; PRF, pontine reticular formation; py, pyramidal tract; RMg, raphe magnus nucleus; RN, red nucleus; rs, rubrospinal tract; RtTg, nucleus reticularis tegmenti pontis; SAI, intermediate white layer; SAP, deep white layer; scp, superior cerebellar peduncle; SGI, intermediate gray layer; SGP, deep gray layer; SGS, superficial gray layer; SO, optic layer; SNr, substantia nigra pars reticulata; sp5, spinal trigeminal tract; ZID, zona incerta, dorsal part; ZIV, zona incerta, ventral part.

In another group of mice (*n* = 3), the period of Dox administration was extended to 21 days, which resulted in strong staining of the cTRNs with anti-GFP immunohistochemistry that filled their distal dendrites. The axons from their cell bodies in the intermediate and deep layers of the right SC (Figure 6A) were labeled all the way to their target areas. Such tracing was difficult to perform in the mice with Dox administration for 7 days. The axons and terminals (Figures 6B,C) of these GFP-positive cTRNs could be found in the right mesodiencephalic junction [e.g., fields of Forel (FF), zona incerta (ZID, ZIV)] (Figure 6Ca), mesencephalic reticular formation (mRt) (Figure 6Cb), nucleus reticularis tegmenti pontis (RtTg) (Figures 6Cc,d), pontine reticular formation (PRF; including the injection site of NeuRet-TRE-EGFP.eTeNT) (Figures 6Cc,d,e), gigantocellular reticular nucleus (Gi) (Figure 6Ce), inferior olivary nuclei (IO) (Figure 6Cf), etc. Thus, in addition to the injection site of the NeuRet vector, positive axons and terminals were found in many other target areas of collaterals that originated from the cTRNs (see Discussion).

### Time course of the dox-on and dox-off periods

**Figure 7:**
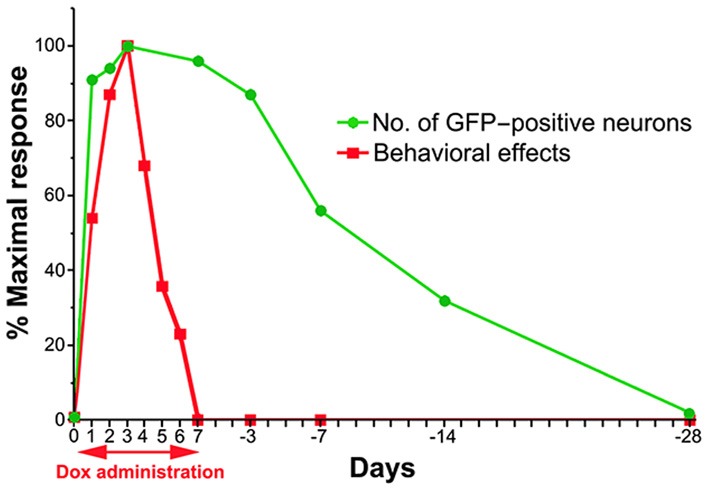
**Schematic drawing of the time course for the emergence and disappearance of blocking effects as measured by the behavioral effects and detectability of anti-GFP immunoreactivity after the onset and offset of Dox administration**.

To compare the time course of the behavioral effects and the appearance of anti-GFP immunoreactivity in cTRNs for the Dox-on and Dox-off periods, the results of the three behavioral tests were normalized to the maximal impairment and averaged. Then, the normalized values were compared to the normalized number of GFP-positive cTRNs during the Dox-on and Dox-off periods (Figure 7). The number of GFP-positive cTRNs increased sharply after the administration of Dox for 1 day, reached its maximum on Dox-on day 3, and was maintained until the end of Dox administration on day 7. Although behavioral impairment was also found from Dox-on day 1, it increased slower than the appearance of GFP-positive cTRNs. In contrast to the number of GFP-positive cTRNs, the behavioral impairment, after reaching its maximal effect on Dox-on day 3, was reduced to near the baseline level on Dox-on days 6–7. After Dox administration was terminated on day 7, the number of GFP-positive cTRNs was gradually reduced and reached the baseline level on Dox-off day −28.

## Discussion

The crossed tectoreticular pathway has been suggested to be involved in the control of orienting responses based on the following evidence: (1) repetitive electrical stimulation of the SC induces contraversive orienting responses (Cowie and Robinson, 1994; Corneil et al., 2002) and that of the medial pontomedullary reticular formation induces ipsiversive orienting responses (Cowie and Robinson, 1994); (2) lesion or reversible inactivation of the SC (Rosenquist et al., 1996; Quaia et al., 1998) or medial pontomedullary reticular formation (Isa and Sasaki, 1988) impairs orienting responses; (3) single unit recordings either from the SC (Sparks, 1975, 1978) or the medial pontomedullary reticular formation (Grantyn and Berthoz, 1987; Isa and Naito, 1995; Isa and Sasaki, 2002) reveal the existence of neurons that show increased firing preceding the orienting response; and (4) anatomically, the medial pontomedullary reticular formation receives massive inputs from the intermediate and deep layers of the contralateral SC (Kawamura and Brodal, 1973; Huerta and Harting, 1982). Thus, there is overwhelming evidence that the tectoreticular pathway plays a major role in the control of orienting responses (Sparks, 1986; Isa and Sasaki, 2002). However, the specific role of cTRNs in gaze has been difficult to resolve. In fact, this difficulty in precisely attributing function to a specific cell population exists for many of the neural systems in the brain. In this regard, the present study, for the first time, showed that pathway-specific and reversible blockade of synaptic transmission worked as efficiently to cause behavioral deficits in mice, as it has in non-human primates (Kinoshita et al., 2012). Moreover, it specifically provides clear evidence that the direct crossed tectoreticular pathway is essential for the execution of orienting responses. Previous pharmacological techniques were not able to dissect this pathway from other tectal output pathways, such as the thalamic projection from the superficial layer, which might be involved in the higher visual processing (May, 2006), and from the uncrossed tecto-reticular pathway, which has been reported to control the avoidance behaviors (Sahibzada et al., 1986; Dean et al., 1989). However, the present method will enable us to pin down the functions of these individual components of the output pathways from the SC in near future.

Finally, we analyzed the methodological details of this technique, especially the time course of the effects of Dox-on and Dox-off with this relatively simple behavioral system. These experiments made use of a fair number of mice, which would be difficult to perform in non-human primates.

## Time course after dox-on

In this study, the behavioral effects of Dox administration could be partially observed as early as 24 h after initiation. This is earlier than observed in our previous study on macaque monkeys, in which the effects appeared on the 2nd day. This may be partly because we used an intraperitoneal injection on the first day to facilitate the quick onset of the effects (Perl et al., 2002). However, a species-related difference cannot be excluded. The effects became maximal on the 2nd and 3rd days after initiation, but gradually became smaller and had mostly disappeared by the 6th day. It is clear that this is not due to the disappearance of the Dox effects on the tetanus toxin production in the doubly infected neurons because the number of GFP-positive cells did not parallel the disappearance of the behavioral effects rather it persisted, as long as Dox was administered continuously. Such a disappearance of the behavioral effects must instead be caused by a compensatory mechanism in the residual pathways. This assumption is partly supported by the observation of a contralateral postural deviation in the turning response at 2 days after Dox-off (Figure 4). It is not clear which neural systems were responsible for such compensation, but it is likely due either to cTRNs, which were spared from double infection by vectors, or to direct corticoreticular pathways or to other unknown pathways involved in the control of visual orienting responses. A combination of these must have taken over the function of the blocked neurons.

## Time course after dox-off

In this study, the behavioral changes were mostly not observed after Dox-offset following 7 days of Dox administration. We noticed a postural deviation to the right side at 2 days after Dox-off, which might be due to a compensatory mechanism established during the Dox-on period. Histological analysis showed that the number of GFP-positive cells gradually decreased after Dox-off, and returned to the baseline level on the 28th day after Dox-off. Furthermore, the 2nd Dox application on the 28th day after Dox-off caused as potent behavioral effects as during the 1st Dox administration. At least a one-month interval was necessary to observe the reversibility of the Dox effect. Since the functional compensation for the loss of transmission through a population of cTRNs may be caused by other compensatory neural circuits, this reversibility suggests that the mouse brain reused the cTRNs that had been blocked, once their transmission was restarted.

## Blockade of transmission through other collaterals of the cTRNs

Our previous study in macaque monkeys (Kinoshita et al., 2012) showed that anti-GFP immunohistochemistry clarified not only the location of the cell bodies of the PNs that expressed EGFP, but also their axonal trajectories. Similarly, a close examination of histological samples after anti-GFP immunohistochemistry revealed that although the number was small, the axons of the cTRNs could be traced from the cell bodies in the intermediate/deep layers of the right SC to their target areas. Axons and terminals could be observed not only in the left medial pontine reticular formation, the injection site of the NeuRet-TRE-EGFP.eTeNT, but also in several nuclei in the mesodiencephalic junction, pons, and medulla, which are targets of the ascending and descending collaterals of tectoreticular neurons (Grantyn and Grantyn, 1982; Huerta and Harting, 1982) (Figure 6). The possibility cannot be excluded that NeuRet-TRE-EGFP.eTeNT had diffused to the right side of the pontine reticular formation from the injection site, and some of these axons and terminals belonged to ipsilaterally projecting tectoreticular neurons. However, this should be a minor occurrence. In fact, the GFP-positive neurons were found exclusively in the caudal and lateral SC, where the cTRNs are located (Figure 5A), despite the fact that injection of AAV2-CMV-rtTAV16 included more medial parts of the SC (Figure 5B). If NeuRet-TRE-EGFP.eTeNT diffused to the right side, the GFP-positive cells should have been found also in more medial and rostral part of the SC where the uncrossed tectoreticular neurons are primarily located (Redgrave et al., 1990), Furthermore, the behavioral effects were clearly those expected from the impairment of cTRNs. In the previous reports (Grantyn and Grantyn, 1982; Isa and Sasaki, 2002), the pontine projection of the cTRNs was described as almost exclusively contralateral. The present report suggests there may be species differences in this regard between rodents and carnivores. As eTeNT was fused with EGFP, it should be transported to the axon terminals in these areas. These results indicate that synaptic transmission to these targets was also blocked, and that the behavioral effects cannot always be ascribed only to the blockade of transmission from the SC to the medial pontine reticular formation, but also to the blockade of signal transmission to all of the target nuclei. To conclude which connection was critical for the observed behavioral effects, another technique, such as optogenetic blockade of synaptic transmission at the nerve terminal (Kaneda et al., 2011), will need to be combined with our approach.

## Other possible uses of this technique

The present results showed that the successful blockade of a particular central pathway with the double viral vector technique could result in behavioral effects in mice. Using a similar highly efficient retrograde gene transfer vector, some of the authors of this article have produced the permanent ablation of a central pathway by using it in combination with an immunotoxin (Inoue et al., 2012). This may represent a good approach for creating animal disease models. However, one of the major advantages of the present technique is its reversibility. Basically, this technique could be applicable to any pathway in the brain. However, when we apply the current technique to other pathways in the brain, it might be necessary to use other serotypes of AAV, because efficiency of transfection of each serotype could vary depending on the target cell types (Blits et al., 2010; Jakovcevski et al., 2010; Markakis et al., 2010). The success of the current method was primarily based on the following factors: (1) development of highly efficient retrograde gene transfer using NeuRet; (2) very efficient amplification of gene expression by the recently developed Tet-ON sequence rtTAV16; and (3) very effective expression of the humanized tetanus neurotoxin eTeNT. Replacing eTeNT with other functional proteins, such as light-sensitive opsins for optogenetic control of neural activity (for reviews see Yizhar et al., 2011; Tye and Disseroth, 2012), may open up a novel direction for neural circuit analysis.

### Conflict of interest statement

The authors declare that the research was conducted in the absence of any commercial or financial relationships that could be construed as a potential conflict of interest.

## References

[B1] Blits B. Derks S. Twisk J. Ehlert E. Prins J. Verhaagen J. (2010). Adeno-associated viral vector (AAV)-mediated gene transfer in the red nucleus of the adult rat brain: comparative analysis of the transduction properties of seven AAV serotypes and lentiviral vectors. J. Neurosci. Methods 185, 257–263 10.1016/j.jneumeth.2009.10.009 19850079

[B2] Corneil B. D. Olivier E. Munoz D. P. (2002). Neck muscle responses to stimulation of monkey superior colliculus. I. Topography and manipulation of stimulation parameters. J. Neurophysiol. 88, 1980–1999 12364523 10.1152/jn.2002.88.4.1980

[B3] Cowie R. J. Robinson D. L. (1994). Subcortical contributions to head movements in macaques. I. Contrasting effects of electrical stimulation of a medial pontomedullary region and the superior colliculus. J. Neurophysiol. 72, 2648–2664 7897481 10.1152/jn.1994.72.6.2648

[B4] Dean P. Redgrave P. Westby G. W. (1989). Event or emergency? Two response systems in the mammalian superior colliculus. Trends Neurosci. 12, 137–147 10.1016/0166-2236(89)90052-0 2470171

[B5] Franklin K. B. J. Paxinos G. (2008). The Mouse Brain: in Stereotaxic Coordinates. 3rd Edn New York, NY: Academic Press

[B6] Grantyn A. Berthoz A. (1987). Reticulo-spinal neurons participating in the control of synergic eye and head movements during orienting in the cat. I. Behavioral properties. Exp. Brain Res. 66, 339–354 10.1007/BF00243309 3595779

[B7] Grantyn A. Grantyn R. (1982). Axonal patterns and sites of termination of cat superior colliculus neurons projecting in the tecto-bulbo-spinal tract. Exp. Brain Res. 46, 243–256 10.1007/BF00237182 7095033

[B8] Huerta M. F. Harting J. K. (1982). Tectal control of spinal cord activity: neuroanatomical demonstration of pathways connecting the superior colliculus with the cervical spinal cord grey. Prog. Brain Res. 57, 293–328 10.1016/S0079-6123(08)64135-7 6296921

[B9] Inoue K. Koketsu D. Kato S. Kobayashi K. Nambu A. Takada M. (2012). Immunotoxin-mediated tract targeting in the primate brain: selective elimination of the cortico-subthalamic“hyperdirect” pathway. PLoS ONE 7:e39149 10.1371/journal.pone.0039149 22761729 PMC3382612

[B10] Isa T. Hall W. C. (2009). Exploring the superior colliculus *in vitro*. J. Neurophysiol. 102, 2581–2593 10.1152/jn.00498.2009 19710376 PMC2777828

[B11] Isa T. Naito K. (1995). Activity of neurons in the medial pontomedullary reticular formation during orienting movements in alert head-free cats. J. Neurophysiol. 74, 73–95 7472355 10.1152/jn.1995.74.1.73

[B12] Isa T. Sasaki S. (1988). Effects of lesion of paramedian pontomedullary reticular formation by kainic acid injection on the visually triggered horizontal orienting movements in the cat. Neurosci. Lett. 87, 233–239 10.1016/0304-3940(88)90454-5 3380346

[B13] Isa T. Sasaki S. (2002). Brainstem control of head movements during orienting; organization of the premotor circuits. Prog. Neurobiol. 66, 205–241 10.1016/S0301-0082(02)00006-0 11960679

[B14] Jakovcevski M. Guo Y. Su Q. Gao G. Akbarian S. (2010). rAAV9–a human-derived adeno-associated virus vector for efficient transgene expression in mouse cingulate cortex. Cold Spring Harb. Protoc. 4, pdb.prot5417 10.1101/pdb.prot5417 20360371

[B15] Kaneda K. Kasahara H. Matsui R. Katoh T. Mizukami H. Ozawa K. (2011). Selective optical control of synaptic transmission in the subcortical visual pathway by activation of viral vector-expressed halorhodopsin. PLoS ONE 6:e18452 10.1371/journal.pone.0018452 21483674 PMC3071716

[B16] Kato S. Inoue K. Kobayashi K. Yasoshima Y. Miyachi S. Inoue S. (2007). Efficient gene transfer via retrograde transport in rodent and primate brains using a human immunodeficiency virus type 1-based vector pseudotyped with rabies virus glycoprotein. Hum. Gene. Ther. 18, 1141–1151 10.1089/hum.2007.082 17944573

[B17] Kato S. Kobayashi K. Inoue K. Kuramochi M. Okada T. Yaginuma H. (2011a). A lentiviral strategy for highly efficient retrograde gene transfer by pseudotyping with fusion envelope glycoprotein. Hum. Gene Ther. 22, 197–206 10.1089/hum.2009.179 20954846

[B18] Kato S. Kuramochi M. Takasumi K. Kobayashi K. Inoue K. Takahara D. (2011b). Neuron-specific gene transfer through retrograde transport of lentiviral vector pseudotyped with a novel type of fusion envelope glycoprotein. Hum. Gene Ther. 22, 1511–1523 10.1089/hum.2011.111 21806473

[B19] Kawamura K. Brodal A. (1973). The tectopontine projection in the cat: an experimental anatomical study with comments on pathways for teleceptive impulses to the cerebellum. J. Comp. Neurol. 149, 371–390 10.1002/cne.901490306 4123505

[B20] Kinoshita M. Matsui R. Kato S. Hasegawa T. Kasahara H. Isa K. (2012). Genetic dissection of the circuit for hand dexterity in primate. Nature 487, 235–238 10.1038/nature11206 22722837

[B21] Kobayashi K. Morita S. Sawada H. Mizuguchi T. Yamada K. Nagatsu I. (1995). Immunotoxin-mediated conditional disruption of specific neurons in transgenic mice. Proc. Natl. Acad. Sci. U.S.A. 92, 1132–1136 10.1073/pnas.92.4.1132 7862648 PMC42652

[B22] Markakis E. A. Vives K. P. Bober J. Leichtle S. Leranth C. Beecham J. (2010). Comparative transduction efficiency of AAV vector serotypes 1-6 in the substantia nigra and striatum of the primate brain. Mol. Ther. 18, 588–593 10.1038/mt.2009.286 20010918 PMC2839442

[B23] May P. J. (2006). The mammalian superior colliculus: laminar structure and connections. Prog. Brain Res. 151, 321–378 10.1016/S0079-6123(05)51011-2 16221594

[B24] Metz G. A. Schwab M. E. (2004). Behavioral characterization in a comprehensive mouse test battery reveals motor and sensory impairments in growth-associated protein-43 null mutant mice. Neuroscience. 129, 563–574 10.1016/j.neuroscience.2004.07.053 15541878

[B25] Montecucco C. Schiavo G. (1994). Mechanism of action of tetanus and botulinum neurotoxins. Mol. Microbiol. 13, 1–8 10.1111/j.1365-2958.1994.tb00396.x 7527117

[B26] Perl A.-K. T. Tichelaar J. W. Whitsett J. A. (2002). Condition gene expression in the respiratory epithelium of mouse. Transgenic Res. 11, 21–29 10.1023/A:1013986627504 11874100

[B27] Pinto L. H. Enroth-Cugell C. (2000). Tests of the mouse visual system. Mamm. Genome. 11, 531–536 10.1007/s003350010102 10886018

[B28] Quaia C. Aizawa H. Optican L. M. Wurtz R. H. (1998). Reversible inactivation of monkey superior colliculus. II. Maps of saccadic deficits. J. Neurophysiol. 79, 2097–2110 9535971 10.1152/jn.1998.79.4.2097

[B29] Redgrave P. Dean P. Westby G. W. (1990). Organization of the crossed tecto-reticulo-spinal projection in rat - I. Anatomical evidence for separate output channels to the periabducens area and caudal medulla. Neuroscience 37, 571–584 10.1016/0306-4522(90)90092-I 1701037

[B30] Rosenquist A. C. Ciaramitaro V. M. Durmer J. S. Wallace S. F. Todd W. E. (1996). Ibotenic acid lesions of the superior colliculus produce longer lasting deficits in visual orienting behavior than aspiration lesions in the cat. Prog. Brain Res. 112, 117–130 10.1016/S0079-6123(08)63324-5 8979824

[B31] Sahibzada N. Dean P. Redgrave P. (1986). Movements resembling orientation or avoidance elicited by electrical stimulation of the superior colliculus in rats. J. Neurosci. 6, 723–733 3958791 10.1523/JNEUROSCI.06-03-00723.1986 PMC6568468

[B32] Smith D. R. Striplin C. D. Geller A. M. Mailman R. B. Drago J. Lawler C. P. (1998). Behavioural assessment of mice lacking D1A dopamine receptors. Neuroscience 86, 135–146 10.1016/S0306-4522(97)00608-8 9692749

[B33] Sooksawate T. Isa K. Isa T. (2008). Cholinergic responses in crossed tecto-reticular neurons of rat superior colliculus. J. Neurophysiol. 100, 2702–2711 10.1152/jn.90723.2008 18753319

[B34] Sooksawate T. Saito Y. Isa T. (2005). Electrophysiological and morphological properties of identified cross tecto-reticular neurons in the rat superior colliculus. Neurosci. Res. 52, 174–184 10.1152/jn.90723.2008 15893578

[B35] Sparks D. L. (1975). Response properties of eye movement-related neurons in the monkey superior colliculus. Brain Res. 90, 147–152 10.1016/0006-8993(75)90690-3 1131686

[B36] Sparks D. L. (1978). Functional properties of neurons in the monkey superior colliculus: coupling of neuronal activity and saccade onset. Brain Res. 156, 1–16 10.1016/0006-8993(78)90075-6 100173

[B37] Sparks D. L. (1986). Translation of sensory signals into commands for control of saccadic eye movements: role of primate superior colliculus. Physiol. Rev. 66, 118–171 3511480 10.1152/physrev.1986.66.1.118

[B38] Tye K. M. Disseroth K. (2012). Optogenetic investigation of neural circuits underlying brain disease in animal models. Nat. Rev. Neurosci. 13, 251–266 10.1038/nrn3171 22430017 PMC6682316

[B39] Watanabe D. Inokawa H. Hashimoto K. Suzuki N. Kano M. Shigemoto R. (1998). Ablation of cerebellar Golgi cells disrupts synaptic integration involving GABA inhibition and NMDA receptor activation in motor coordination. Cell 95, 17–27 10.1016/S0092-8674(00)81779-1 9778244

[B40] Wurtz R. H. Albano J. E. (1980). Visual-motor function of the primate superior colliculus. Annu. Rev. Neurosci. 3, 189–226 10.1146/annurev.ne.03.030180.001201 6774653

[B41] Yizhar O. Fenno L. E. Davidson T. J. Mogri M. Deisseroth K. (2011). Optogenetics in neural systems. Neuron 71, 9–34 10.1016/j.neuron.2011.06.004 21745635

